# Exploring the impact of sleep on emotional and physical well-being in professional cricketers: a cohort study over an in-season training period

**DOI:** 10.3389/fspor.2024.1389565

**Published:** 2024-06-06

**Authors:** S. Grewal, R. T. Theijse, G. Dunlop, D. F. P. van Deurzen, M. P. J. van den Bekerom, R. J. M. Klautz, R. P. Lefebvre, D. Munsami, N. Grewal

**Affiliations:** ^1^ Department of Orthopaedic Surgery, Shoulder and Elbow Unit, Onze Lieve Vrouwe Gasthuis (OLVG), Amsterdam, Netherlands; ^2^ Department of Cardiothoracic Surgery, Amsterdam University Medical Center, Amsterdam, Netherlands; ^3^ Physiotherapist, Royal Dutch Cricket Association, Nieuwegein, Netherlands; ^4^ Faculty of Behavioral and Movement Sciences, Department of Human Movement Sciences, Amsterdam Movement Sciences, Vrije Universiteit Amsterdam, Amsterdam, Netherlands; ^5^ Department of Cardiothoracic Surgery, Leiden University Medical Center, Leiden, Netherlands; ^6^ Department of Anatomy and Embryology, Leiden University Medical Center, Leiden, Netherlands

**Keywords:** sleep, emotional well-being, physical well-being, athletes, cricket

## Abstract

**Background:**

Professional athletes navigate a multitude of unique challenges associated to sport-specific factors (e.g., training, travel and competition) and non-sport factors (e.g., performance pressure, stress and anxiety) that can interfere with healthy sleep behaviors. Sleep plays a key role in proper biopsychosocial development as well as short- and long-term biological, physical, psychological, and cognitive health. As poor sleep quality is known to impair proper brain function, this study aimed to investigate the effect of sleep quality on a professional athlete's ability to train, recover, and perform, as well as their overall emotional and physical well-being.

**Methods:**

A cohort study was performed in 40 professional male cricket athletes from the Dutch national cricket team (mean age 26.5 ± 5.1 years). The athletes were monitored across a 22 weeks in-season training period. Sleep quality and overall emotional and physical well-being were assessed using daily sleep diaries and questionnaires which scored the readiness to train, stress levels, fatigue, muscle soreness and flu symptoms respectively. Quality of sleep and subsequent association with the consecutive elements of the well-being questionnaire were assessed through statistical using the student *t*-test and clinical differences with the methodology of Osoba and colleagues: <5% “no change”, 5%–10% “little change”; 10%–20% “moderate change”; and >20% “very much change”.

**Results:**

The results demonstrated that the professional athletes assessed their sleep quality as average with a mean score of 3.4 out of 5. Lower perceived quality of sleep (<75th percentile) was correlated with a decreased readiness to train (mean score 3.2 [IQR: 3.0–4.0] vs. 3.5 [IQR: 3.0–5.0]; *P* < 0.001) and increased extent of muscle soreness (2.7 [IQR: 2.0–3.0] vs. 2.3 [IQR: 2–3]; *P* < 0.001), stress level (mean score 2.3 [IQR: 2.0–3.0] vs. 1.9 [IQR: 1.0–2.0]; *P* < 0.001) and perceived fatigue (mean score 2.9 [IQR: 2.0–3.0] vs. 2.3 [IQR: 2.0–3.0]; *P* < 0.001). Likewise, in patients with lower perceived quality of sleep, the proportion of players presenting with flu symptoms increased over 4-fold (4.1% vs. 17%; *P* < 0.001).

**Conclusions:**

This study highlights that good sleep quality positively influences the overall emotional and physical well-being of professional athletes. Our results emphasize the importance of targeted sleep interventions to improve sleep quality and subsequently optimize psychological and physiological wellness.

## 1 Introduction

In the high-pressure world of professional sports, where split-second decisions and peak physical performance can make all the difference between victory and defeat, the importance of sleep cannot be overstated. Professional cricketers, like athletes in any sport, rely on a combination of talent, skill, and rigorous training to excel on the field. However, amidst the spotlight of stadiums and the intensity of competition, one often overlooked factor plays a crucial role in determining an athlete's success: sleep.

Sleep is an essential human behavior that plays an important role in one's physiological function, social interactions, and athletic performance (1–4). Sleep has an adaptive and restorative function both within the brain as well as the physiological processes in the body (4, 5) all of which are vital for athletes to excel in their sport. In the sport of cricket, where matches can last for several days and involve intense physical and mental exertion, sleep is particularly important.

In certain sports other than cricket, such as football or basketball, a growing body of literature has demonstrated a positive relationship between sleep and optimal performance (6), whereas compromised sleep quantity and quality has been shown to adversely affect health, increase risk of injury and impair athletic performance, learning and memory (6–9). Studies in the general population further show that habitually sleeping <7 h/night increases vulnerability to develop respiratory infections (10). By prioritizing sleep and implementing strategies to improve sleep quality and quantity, athletes can enhance their overall health, performance, and longevity in the sport. Coaches, sports scientists, and support staff should recognize the importance of sleep in athlete development and incorporate sleep hygiene education and support into training and recovery programs.

Professional cricketers represent a unique population that face numerous obstacles in acquiring enough and good quality sleep. In the demanding strenuous training regimes, performance pressure, anxiety and/or high levels of stress related to competitions and extensive (long distance) traveling necessities, sleep often tends to be put off in favor of other activities (6, 11). From the serene calm of pre-match preparation to the adrenaline-fueled drama of game day, sleep thus impacts every aspect of a cricketer's performance, both physically and mentally. Yet, despite its significance, sleep remains a widely underappreciated aspect in professional cricket and to the best of the authors' knowledge, there is no specific research investigating the influence of sleep quality in professional athletes (12–14).

In this article, we therefore focus on the impact of sleep on the emotional and physical well-being in professional cricketers. We hypothesize that good sleep quality positively affects the overall emotional and physical well-being of professional athletes. Players of the national Dutch cricket team are followed throughout an in-season training phase to examine the relationship between sleep quality and measures of emotional and physical well-being including the *readiness to train, general muscle soreness, general stress level, fatigue level and flu symptoms*.

## 2 Material and methods

All players which are part of the national Dutch cricket team training for the international World Cup Cricket held in India in 2023, were included in the study, no sample size calculation was performed. Players who could not finish the 22 weeks follow-up study and/or were not going to be a part of the cricket world cup were excluded from the study. This led to an inclusion (100%) of forty male professional cricket athletes (mean age 26.5 ± 5.1 years) in the study. The participating cricketers were all classified as Tier 5 *World Class* according to the Participant Classification Framework described bij McKay et al. (15). Participants were recruited from 16 April 2023 till 15 August 2023. Written informed consent was obtained from all participants. All study protocols were approved by the Human Research Ethics Committee from the Leiden University Medical Center (Leiden-Den Haag-Delft (METC-LDD, ref: B21.051/MS/ms).

An observational study design was utilized for the current investigation. The study was performed during an in-season training period in the Netherlands. Each week within this phase comprised of 3–4 training days, which consisted of skills and strength training sessions and 1–2 game days. The training hours were on average 3–4 h and the training intensity RPE 5–6/10. All players were trained by the same head coach, a strength and conditioning coach and a physiotherapist. Each morning prior to the start of the training, for 22 consecutive weeks, all consenting players completed the sleep diary and psychological and physical well-being questionnaires to assess participants' sleep quality and overall mental and physical well-being respectively. The questionnaire applied in the study was derived from the Hooper index (16). Participants were monitored daily for noting self-reported *sleep quality, readiness to train, general muscle soreness, general stress level, and fatigue level* (11, 13, 17, 18). The questionnaire used a 1–5 Likert scale. For *sleep quality* and *readiness to train* 1 represented a low score (e.g., very poor) and 5 represented a high score (e.g., very good). For the remaining questions (being *general muscle soreness, general stress level and fatigue*) a lower score indicated less muscle soreness, stress and fatigue and greater scores indicated extreme muscle soreness, stress and fatigue. *Flu symptoms* were assessed as being present or not (10). Correlation between sleep quality and all elements of the well-being questionnaire were assessed individually. The questionnaire was a routine element of the training schedule, checked by the leading physiotherapist during each session.

### 2.1 Statistical analysis

Data analyses were performed with RStudio: Integrated Development Environment for R (Software Version 1.3.1093, Boston, MA). Descriptive statistics were compiled to summarize characteristics. Binary variables are presented as percentages and frequencies, and numerical variables as means with corresponding interquartile ranges (IQR). Quality of sleep and subsequent association with the consecutive elements of the well-being questionnaire were investigated by assessment of statistical and clinical differences using the student *t*-test. The clinical difference was assessed with the methodology of Osoba and colleagues: <5% “no change”, 5%–10% “little change”; 10%–20% “moderate change”; and >20% “very much change” (19). Ideally, a generalized linear mixed model was fitted to examine the relationship between quality of sleep and corresponding wellness scores, while accounting for other predictors. However, due to sample-size fitting of a generalized linear mixed model may lead to unreliable results and will therefore not be performed. For all tests, statistical significance was defined as a two-tailed value of *P* < 0.050.

## 3 Results

In total, 40 5-tier world-class cricketers were evaluated during the 22 weeks in-season training period prior to the 2024 world cup. The response rate was 100% without dropouts or missing results.

*Sleep quality, muscle soreness, readiness to train, stress level and fatigue* were reported on a Likert-scale of 5 points (1–5 points), whereas *flu symptoms were* assessed as a binary variable (i.e., being present or not).

**Figure 1:**
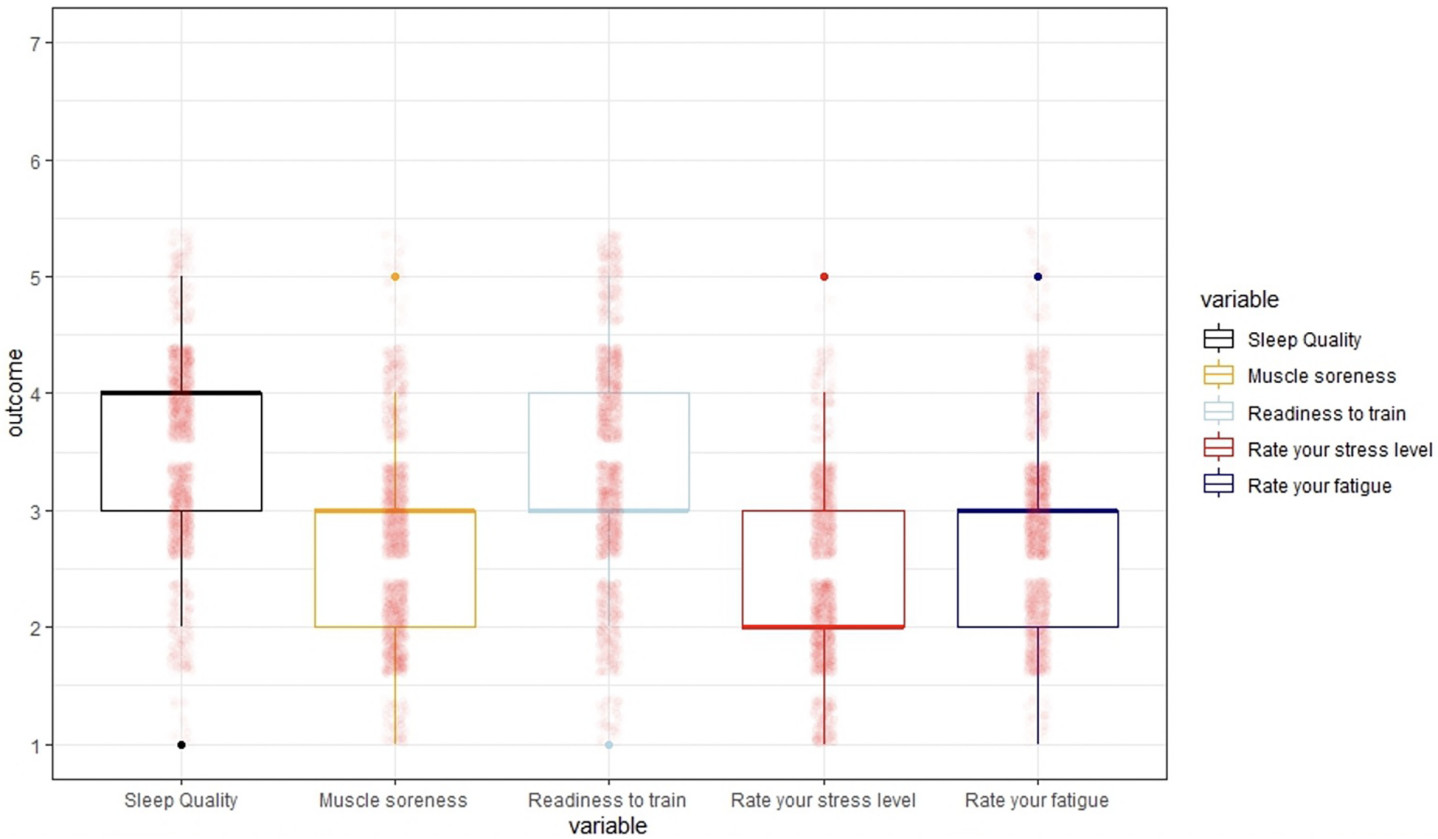
Overview of study outcomes. Box-and-whisker plot summary statistics of average sleep quality, muscle soreness, readiness to train, stress- and fatigue scores. The boxes represent the middle 50% of the observed values, dots are outliers (the black box represents the variable sleep quality, the yellow box muscle soreness, the blue box readiness to train, the red box stress level and the purple box fatigue).

Overall, sleep quality was scored as average by the athletes (mean score 3.4), which was in line with their readiness to train (mean score 3.3). The athletes indicated to have slight muscle soreness (mean score 2.6), stress (mean score 2.3) and fatigue (mean score 2.8), as outlined in Table 1. See Figure 1 for a Box-and-whisker plot summarizing al 5 wellness scores. Fifty percent of the athletes scored sleep quality and readiness to train between 3.0 and 4.0. Muscle soreness, and stress- and fatigue levels ranged from 2.0 to 3.0.

**Table 1:** Outcomes of all wellness scores and flu symptoms.

Questions	Cohort scores
Can you rate your Quality of Sleep on scale of 1–5?	3.4 [3.0–4.0]
Can you rate the extent of Muscle Soreness on scale of 1–5?	2.6 [2.0–3.0]
Can you rate your Readiness to train on scale of 1–5?	3.3 [3.0–4.0]
Can you rate your Stress level on a scale of 1–5?	2.3 [2.0–3.0]
Can you rate your Fatigue on scale of 1–5?	2.8 [2.0–3.0]
Do you have any flu symptoms (yes/no)?	326 (15.9% yes)

Mean scores [IQR], all questions are ranged from 1 to 5. For sleep quality and readiness to train 1 represented a low score (e.g., very poor) and 5 represented a high score (e.g., very good) for the remaining questions (being general muscle soreness, general stress level and fatigue) a lower score indicated less muscle soreness, stress and fatigue and greater scores indicated extreme muscle soreness, stress and fatigue.

In total, 15.9% of the athletes had flu symptoms during the study period (Table 1).

**Figure 2:**
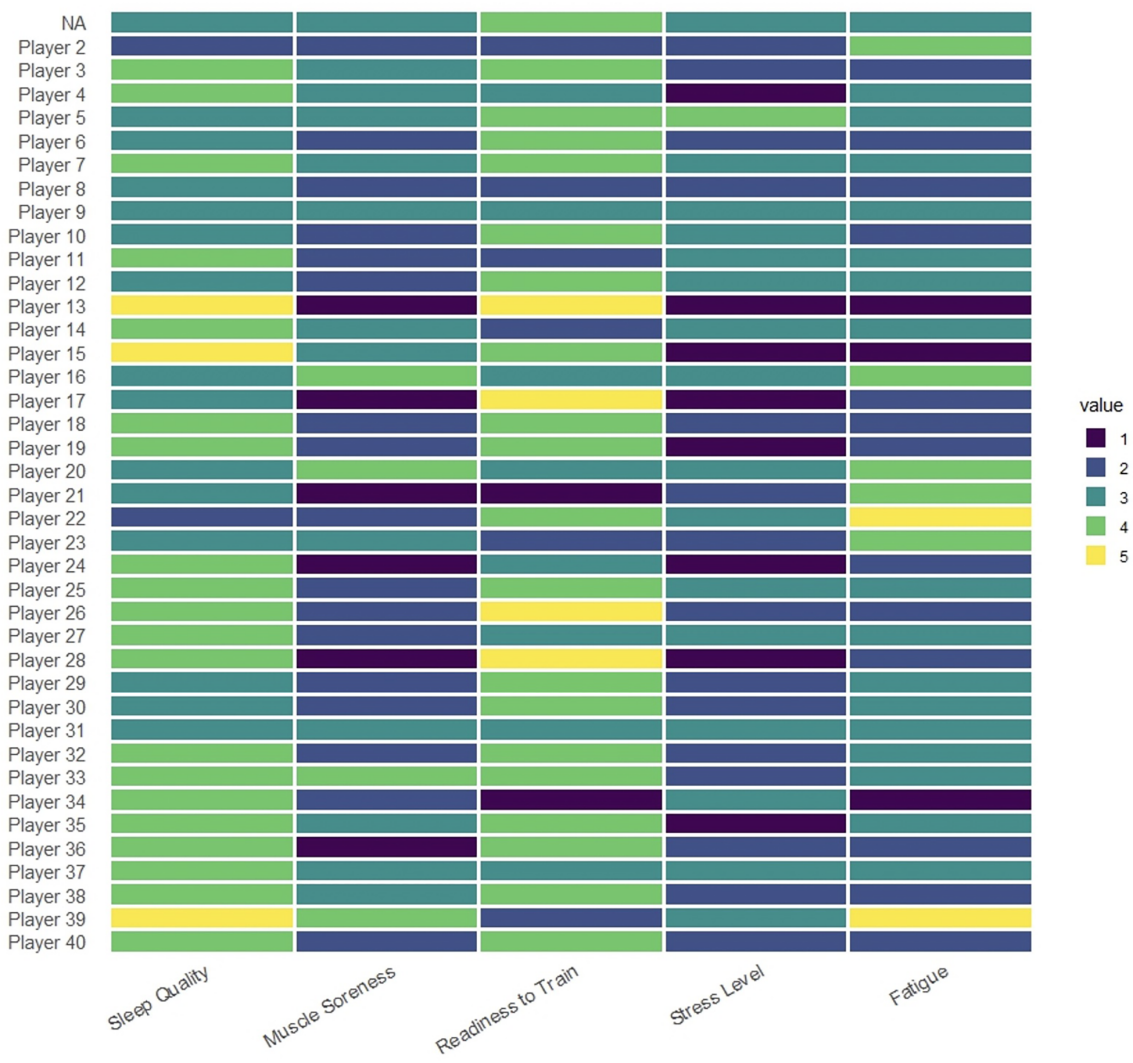
Relative distribution of outcomes for each wellness score. The relative distribution of sleep quality, muscle soreness, readiness to train, stress- and fatigue scores are shown for each participant.

When assessing the individual results, it is apparent that a better sleep quality enhances the readiness to train and decreases muscle soreness, stress and fatigue levels (Figure 2). Better sleep quality of each player is associated with an enhanced readiness to train and decreased muscle soreness, stress and fatigue level. When comparing the sleep quality below and above the 75th percentile, a significant difference was objectified, whereby muscle soreness increased (2.7 vs. 2.3; *P* < 0.001) while readiness to train (*P* < 0.001), stress levels (*P* < 0.001), fatigue (*P* < 0.001) and symptoms of flu (*P* < 0.001) increased respectively (Table 2). Each point increase towards better quality of sleep, is associated with a significant increase in readiness to train and reduction in muscle soreness and stress- and fatigue levels (*P* < 0.001). Additionally, significant less flu symptoms were reported in athletes with good quality of sleep (*P* < 0.001) (Table 3).

**Table 2:** Impact of sleep quality on wellness scores.

Questionnaire	Sleep quality below the 75th percentile	Sleep quality above the 75th percentile	Δ score	Subjective significance	*P*-value
	(*n *= 1883)	(*n *= 172)			
Sleep quality	≤3.0	≥5.0	–	–	–
Muscle soreness	2.7 [2.0–3.0]	2.3 [2.0–3.0]	−0.4	Moderate change	<0.001
Readiness to train	3.2 [3.0–4.0]	3.5 [3.0–5.0]	+0.3	Little change	<0.001
Stress level	2.3 [2.0–3.0]	1.9 [1.0–2.0]	−0.4	Moderate change	<0.001
Fatigue	2.9 [2.0–3.0]	2.3 [2.0–3.0]	−0.6	Very much change	<0.001
Flu symptoms	319 (17%)	7 (4.1%)	−12.9	Very much change	<0.001

A significant difference in the wellness score results were seen when comparing the sleep quality below and above the 75th percentile.

**Table 3:** Wellness scores stratified for each sleep quality indicator.

Questionnaire	Sleep quality 1	Sleep quality 2	Sleep quality 3	Sleep quality 4	Sleep quality 5	*P*-value
	(*n *= 46)	(*n *= 212)	(*n *= 759)	(*n *= 866)	(*n *= 172)	
Muscle soreness	2.7 [2.0–3.0]	3.0 [2.0–3.0]	2.7 [2.0–3.0]	2.5 [2.0–3.0]	2.3 [2.0–3.0]	<0.001
Readiness to train	2.1 [1.0–3.0]	2.7 [2.0–3.0]	3.2 [3.0–4.0]	3.5 [3.0–4.0]	3.5 [3.0–5.0]	<0.001
Stress level	2.3 [2.0–3.0]	2.3 [2.0–3.0]	2.4 [2.0–3.0]	2.2 [2.0–3.0]	1.9 [1.0–2.0]	<0.001
Fatigue	3.6 [3.0–4.8]	3.5 [3.0–4.0]	2.9 [2.0–3.0]	2.6 [2.0–3.0]	2.3 [2.0–3.0]	<0.001
Flu symptoms	6 (13%)	41 (19%)	133 (18%)	139 (16.1%)	7 (4.1%)	<0.001

Each point increase towards better quality of sleep, resulted in a significant increase in readiness to train and reduction in muscle soreness and stress- and fatigue levels (*P* < 0.001). Significantly less flu symptoms were observed in athletes with good quality of sleep (*P* < 0.001).

## 4 Discussion

This study evaluated the relationship between quality of sleep and the mental-and physical well-being among professional cricketers. The main finding of this study is that increased quality of sleep is significantly related with lower levels of muscle soreness, stress and fatigue and results in higher readiness to train. Additionally, the professional athletes significantly observed less flu like symptoms with good quality of sleep. By contrast, poor quality of sleep was related with significantly high levels of negative psychological and physical health.

Professional cricketers often face significant physical and emotional stress due to the demanding nature of their sport. Physical stresses include (1) rigorous training schedules with strength and conditioning exercises, skill drills, and match simulations, which can lead to muscle fatigue, joint strain, and the risk of overuse injuries; (2) match intensity: Cricket matches, especially in formats like Test cricket and limited-overs internationals, can be physically demanding, requiring athletes to maintain focus and intensity for extended periods. Cricketers may experience physical exhaustion, dehydration, and heat stress during intense matches played in challenging conditions; (3) travel fatigue: professional cricketers often travel extensively for domestic and international competitions, which can lead to jet lag, disrupted sleep patterns, and fatigue. Long flights, frequent time zone changes, and irregular schedules can take a toll on the body and affect performance on the field.

Emotional stressors professional cricketers must deal with are (1) performance pressure: professional cricketers face immense pressure to perform at their best in every match. Expectations from fans, coaches, teammates, and sponsors can create significant psychological stress, leading to anxiety, self-doubt, and fear of failure; (2) public scrutiny: cricketers operate in the public eye, with their performances scrutinized by media, fans, and critics. Negative media coverage, social media scrutiny, and public criticism can contribute to stress and mental strain, affecting confidence and self-esteem; (3) injury concerns: injuries are a common occurrence in cricket, and the fear of getting injured or aggravating existing injuries can be a source of emotional stress for cricketers. Injuries not only impact physical performance but also disrupt training schedules and affect mental well-being; (4) personal sacrifices: the demands of professional cricket often require players to make significant personal sacrifices, such as spending time away from family and friends, missing important events, and adhering to strict dietary and lifestyle restrictions. These sacrifices can lead to feelings of isolation, loneliness, and homesickness.

Adequate rest, recovery, and downtime are essential for cricketers to recharge physically and emotionally. Balancing training and competition with sufficient rest periods can help prevent burnout and reduce the risk of injury. This study aimed at exploring the relationship between adequate sleep quality and self-reported measures of emotional and physical wellbeing.

The findings of our study reconfirm the prevalence of poor sleep quality in professional athletes. Drew et al. reported a poor quality of sleep in more than 50% of the athletes from 11 different sports participating in the Olympic Games (20). A strong association has been reported between sleep quality and an athlete's overall well-being, in sports such as football and rugby (21). Despite the physical and emotional challenges in the world of cricket, specific research on cricketers is somewhat limited and this study is, to our knowledge, the first till date investigating the association between sleep quality and general psychological and physical well-being of professional cricketers (11, 13, 17). Across the current study, 75th percentile of the professional cricketers reported a sleep quality score below 3, demonstrating a high degree of poor sleep quality within the cohort.

The subjective methods to collect sleep data with sleeping diaries, might have limited the interpretation of the acquired data, it did aid in understanding inter-individual differences among the players. Notably, there were large inter-individual differences in sleep quality in the cricket players which has also been reported in other team-sports athletes (ref). The large variability indicates that the sleep quality might have a greater effect on some athletes compared to others, emphasizing the importance of individualized sleep monitoring.

In this study, poor sleepers reported significantly greater stress compared to good quality sleepers. On the other hand, increased levels of stress, which are often reported in pre-competition athletes, are also associated with poor sleep (22). The bidirectional relationship of poor sleep quality and increased stress is widely acknowledged (6). Therefore, stress should be taken into consideration when interpreting sleep behavior data, due to the transactional relationship between these two variables. In conclusion, poor sleep health is likely to lead to worse competitive performance through indirect pathways, such as insufficient and ineffective training, increased frequency of physical injury and illness, and degraded mental health in every professional athlete. Although the obtained longitudinal data are a strength of this study, further studies are needed to clarify the bidirectional relation between quality of sleep and the psychological and physical well-being of professional athletes in order to develop appropriate plans of action for optimization. There is a need for future research in other professional cricket teams to develop tailored and effective interventions for improving sleep health among professional athletes and evaluate how enhancing sleep health impacts mental health and subsequently game performance. Ultimately, by fostering a culture of sleep awareness and prioritization, professional cricketers can optimize their performance and well-being on and off the field.

## 5 Study strengths and limitations

The strengths of the current study are that the cohort included professional athletes from the Dutch national cricket team. Additionally, the study was performed during a complete in-season training period prior to the World Cup. The most evident limitation of this study was only using subjective methods to collect sleep data, future investigations should aim to use both subjective and objective measures to obtain a more comprehensive understanding. Secondly, the study was performed in a single national cricket team; therefore, the results may not be generalizable to professional athletes.

## 6 Conclusion

In conclusion, sleep quality is negatively associated with overall well-being in professional cricketers. Considering the paramount role of sleep health in the training, recovery, performance and overall well-being of professional athletes, the unique challenges faced by professional athletes that negatively impact sleep health, and the high prevalence of sleep problems among professional athletes, there is a clear need for tailored strategies to enhance sleep health in professional athletes.

## Data Availability

The original contributions presented in the study are included in the article/Supplementary Material, further inquiries can be directed to the corresponding author.
